# Exploring non-viral methods for the delivery of CRISPR-Cas ribonucleoprotein to hematopoietic stem cells

**DOI:** 10.1186/s13287-024-03848-4

**Published:** 2024-07-29

**Authors:** Zahra Molaei, Zahra Jabbarpour, Azadeh Omidkhoda, Naser Ahmadbeigi

**Affiliations:** 1 Hematology and blood transfusion science department, School of Allied Medical Sciences, Tehran University of Medical Sciences, Tehran, Iran; 2 School of Pharmacy & Bioengineering, Guy Hilton Research Centre (GHRC), Keele University, Staffordshire, ST4 7QB UK; 3 Gene Therapy Research Center, Digestive Disease Research Institute, Tehran University of Medical Sciences, Tehran, Iran

**Keywords:** Hematopoietic stem cell, CRISPR-Cas System, Genome editing, Genetic therapy, Delivery strategies

## Abstract

Gene manipulation of hematopoietic stem cells (HSCs) using the CRISPR/Cas system as a potent genome editing tool holds immense promise for addressing hematologic disorders. An essential hurdle in advancing this treatment lies in effectively delivering CRISPR/Cas to HSCs. While various delivery formats exist, Ribonucleoprotein complex (RNP) emerges as a particularly efficient option. RNP complexes offer enhanced gene editing capabilities, devoid of viral vectors, with rapid activity and minimized off-target effects. Nevertheless, novel delivery methods such as microfluidic-based techniques, filtroporation, nanoparticles, and cell-penetrating peptides are continually evolving. This study aims to provide a comprehensive review of these methods and the recent research on delivery approaches of RNP complexes to HSCs.

## Introduction

The Clustered Regularly Interspaced Short Palindromic Repeats (CRISPR) locus, a 1664-nucleotide sequence, was first discovered in E. coli in 1987 [1]. Although various studies have explored this gene locus and similar loci found in different bacteria and archaea species [2–5], the primary function of this locus remained unknown until 2007. The exploration of genes adjacent to the CRISPR locus [6] and the identification of foreign viral DNA within the CRISPR locus spacers prompted the hypothesis that this locus functions as an adaptive immune system in bacteria and archaea, defending against viral invasions [7, 8]. This hypothesis was confirmed by the Barrangou Lab in 2007 [9]. Due to its ability to precisely target and cut specific DNA sequences, the CRISPR-Cas system was recognized as a genome editing tool in 2012 [10]. This system performs much better than tools previously used, such as meganucleases, zinc finger nucleases (ZFNs), and transcription activator-like effector nucleases (TALENs). Its design and implementation are much simpler, and it has broader applications [11].

The CRISPR-Cas9 system, Type II from S. pyogenes, is the most common variant among this system’s two types and six subtypes [12]. It creates specific double-strand breaks (DSBs) and facilitates targeted genome editing [13]. This system comprises an RNA-guided endonuclease, Cas9 endonuclease, which is directed to the target sequence by a dual guide RNA consisting of CRISPR RNA (crRNA) and transactivating RNA (tracrRNA), forming a sequence-specific endonuclease for cleaving the target DNA [14]. Dual tracrRNA: crRNA can be synthesized into a single guide RNA (sgRNA). The initial 20 nucleotides at the 5’ end complement the target DNA sequence and connect to it via Watson-Crick base pairing. At the 3’ end of this sgRNA, a double-stranded structure acts as a scaffold for connecting the sgRNA to Cas9 [10].

The Cas9 enzyme, guided by RNA (gRNA), identifies the target sequence in DNA and creates a double-strand break (DSB) in the DNA. Creating a DSB is the initial step in genome editing, and cells respond to it through two different mechanisms: nonhomologous end-joining (NHEJ) and homology-directed repair (HDR). NHEJ can lead to the efficient introduction of insertion/deletion mutations (indels) of various lengths. HDR-mediated repair can introduce specific point mutations or insert desired sequences through recombination of the target locus with exogenously supplied DNA ‘donor templates’ [15]. A significant hurdle in applying CRISPR/Cas9 technology lies in off-target events, unintended edits within the host’s DNA, that lead to alterations at locations other than the intended target sites. Off-target effects are particularly problematic in complex organisms with extensive genomes, such as humans, who are more likely to experience these unintended mutations than organisms with simpler genetic makeups [16]. Moreover, the success of gene editing does not only hinge on the accuracy of the Cas9 enzyme and its associated guide RNA; the method used to introduce these elements into the organism dramatically affects the outcome. Identifying dependable and harmless delivery techniques is crucial for integrating this technology into medical treatments [17].

CRISPR/Cas9 gene editing allows for a wide range of precise modifications, including gene insertion, gene knockout, alteration of individual bases, regulation of gene transcription, and comprehensive genetic screening [18]. The application of CRISPR/Cas9 technology has expanded across various fields, fundamentally reshaping research on the pathological mechanisms and therapeutic strategies for different diseases, such as Alzheimer’s disease (AD) [19], AIDS [20], autoimmune diseases [21], cystic fibrosis [22, 23], Huntington’s chorea disorder [24], Duchenne muscular dystrophy [25], chronic granulomatous disease [26], retinitis pigmentosa [27], hemophilia [28, 29], sickle cell disease (SCD) [30–32] and thalassemia [31, 33, 34].

Hematopoietic stem cell transplantation (HSCT) is a key treatment strategy for various blood disorders and cancers, offering the chance to rebuild the hematopoietic system. In cases like thalassemia major in children or SCD, HSCT from a closely matched donor is often the only definitive cure for defective red blood cell production [35]. However, finding a donor with compatible human leukocyte antigen (HLA) markers can be challenging, and patients may face risks such as graft-versus-host disease (GVHD) and infections due to weakened immunity [36]. Given these challenges, using the patient’s genetically modified hematopoietic stem cells (HSCs) (autologous HSCT) is an effective and safer alternative. HSCs present a particular challenge for gene editing due to their quiescent nature and distinct cellular properties, making them resistant to standard transfection approaches like viral vectors or chemical methods [37]. Researchers are therefore exploring innovative strategies to enhance gene delivery efficiency to HSCs. Achieving precise targeting of the CRISPR/Cas9 system to specific cell types and tissues is another significant hurdle, essential for the real-world application of gene therapies [38].

Hematopoietic stem cells play a crucial role in regenerative medicine due to their unique ability to self-renew and differentiate into various blood cell lineages, making them invaluable for therapeutic applications. HSCs are not only essential for the lifelong maintenance of blood homeostasis but also hold significant promise for treating a variety of hematological disorders and immune deficiencies through transplantation. However, as noted by Montazersaheb et al., the aging of HSCs and the mechanisms of autophagy in these cells are critical factors influencing their efficacy and safety in clinical applications. Aging HSCs exhibit diminished regenerative capacity and an increased propensity for malignancies, necessitating interventions to rejuvenate these cells and maintain their functionality [39]. Additionally, understanding the role of autophagy—a cellular degradation and recycling process—in HSC transplantation can provide insights into improving the outcomes of these procedures. Strategies to enhance autophagy may protect HSCs from stress and improve their survival and engraftment post-transplantation. By integrating knowledge of these cellular and molecular mechanisms, advancements in CRISPR-Cas9 technology and non-viral delivery systems for gene editing of HSCs could significantly enhance the therapeutic potential of HSCs in regenerative medicine [40].

While previous reviews have covered the broader topic of non-viral delivery systems for CRISPR-Cas9-based genome editing [41–46], there remains a significant gap in the literature regarding the specific challenges and advancements in delivering RNP to hematopoietic stem cells. HSCs possess unique characteristics and therapeutic potential that distinguish them from other cell types, necessitating a focused review on this topic. Our paper addresses this gap by exclusively examining non-viral delivery methods tailored for RNP delivery to HSCs, providing the most recent updates and a detailed comparison of the strengths and weaknesses of each method. This targeted approach aims to offer valuable insights and guidance for researchers working on gene therapy applications involving HSCs.

## Different formats of delivering CRISPR-Cas9 components to cells

**Fig. 1:**
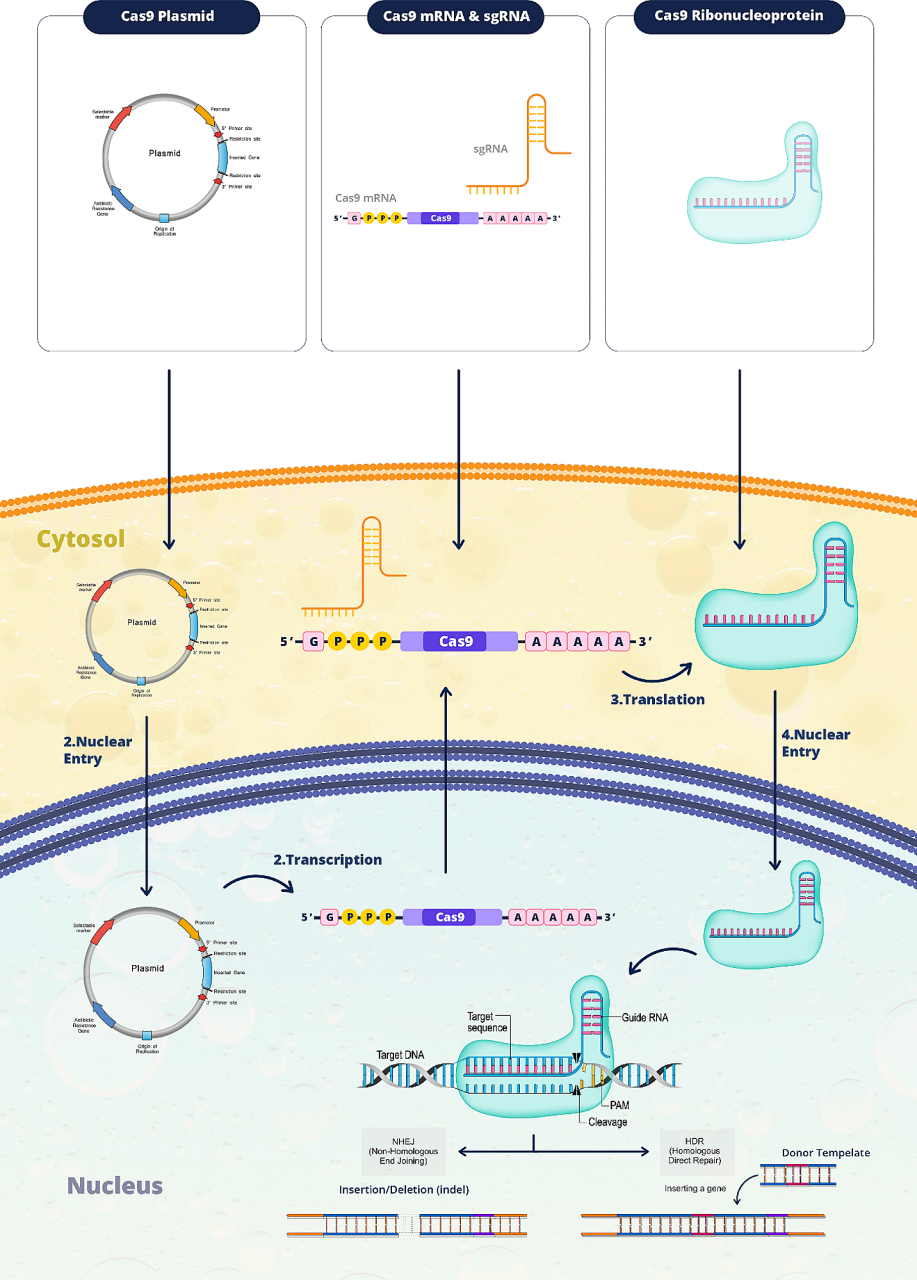
Comparison of different biomolecular CRISPR/Cas9 formats. In the process of delivering plasmid DNA, it is essential that the plasmid reaches the nucleus. Here, the transcription mechanism activates, converting the gene into gRNA and Cas9 mRNA. Following this, in the cytoplasm, Cas9 mRNA undergoes translation to produce Cas9 protein. Subsequently, both gRNA and Cas9 protein are transported back into the nucleus, where the CRISPR mechanism can enact its effect on the targeted genomic DNA. Alternatively, for Cas9 mRNA delivery, the cargo must be released into the cytosol. This allows for the translation of mRNA into Cas9 protein to occur. Notably, the Ribonucleoprotein (RNP) delivery method offers instantaneous results compared to other strategies. By bypassing the translation and transcription processes, gene editing can commence immediately upon delivery. (This figure was created by the authors using Adobe Photoshop software)

The CRISPR-Cas9 system can be introduced into cells using: DNA, RNA, and protein (Fig. 1). 2.1. Introducing Genes with Plasmid DNA.

Employing plasmid DNA entails inserting one or two plasmids directly into the nucleus to transport the blueprints for the Cas9 enzyme and the sgRNA. While this method benefits from the plasmid’s inherent stability and ease of use, it has its pitfalls. The complex journey of these plasmids to the nucleus and their subsequent conversion into proteins is fraught with challenges, notably the risk of accidental integration into the cell’s DNA, which heightens the chances of unintended genetic alterations [39].

### Employing mRNA and sgRNA for Cas9

Moving away from plasmid DNA, the alternative of utilizing mRNA to deliver the instructions for Cas9 presents a streamlined and ostensibly safer method. This process circumvents the complexities of nuclear entry since protein creation occurs in the cytoplasm and avoids permanent incorporation into the cell’s DNA. The primary obstacle here is the fragility of mRNA, which is prone to degradation. To mitigate this, the mRNA is chemically modified once inside the cell to increase its longevity and ensure the successful production of proteins [40].

### Cas9 and sgRNA via Ribonucleoprotein complex (RNP)

This innovative strategy involves delivering the Cas9 enzyme and sgRNA as a pre-formed complex in vitro. This route offers a significant advantage by eliminating the risk of becoming a permanent part of the cell’s DNA. The transient nature of the Cas9 enzyme also reduces the likelihood of triggering an immune response and decreases the chance of editing genes unintentionally [41, 42]. Notably, RNPs have been shown to achieve more precise and efficient gene editing outcomes, making them preferable compared to the previously mentioned DNA and mRNA vehicles. Nevertheless, mass-producing Cas9 and sgRNA for this method remains a daunting challenge (43).

## Strategies for RNP delivery to hematopoietic stem cells

**Fig. 2:**
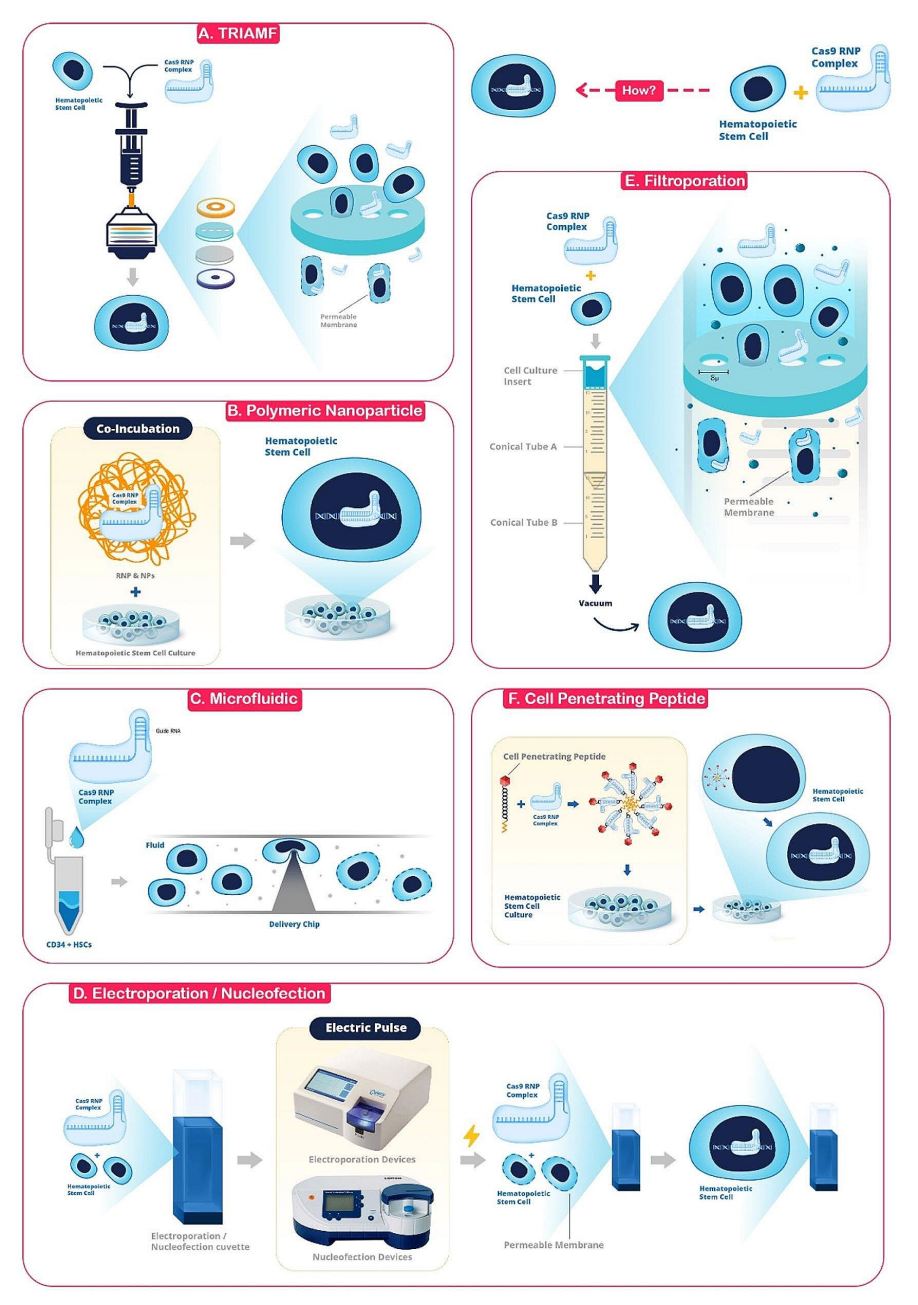
Schematic of strategies for delivering Cas9 RNP complex to HSCs. **a**. TRIAMF Filtroporation Device. Composed of a silicone washer, stainless-steel mesh, polycarbonate filter membrane, and PTFE washer. The syringe reservoir contains the RNP and HSC mixture, which is propelled through the membrane using nitrogen pressure into a culture plate. **b**. Polymer Nanoparticles (PNPs) for RNP Delivery. The figure illustrates the use of PNPs to deliver RNP complexes. The depicted polymeric structures have been proven safe and efficient for delivering CRISPR-Cas9 system components, with controllable speed, timing, and location of delivery. **c**. Microfluidic-Based Nano-Blade Chip. This figure showcases the Nano-Blade chip, constructed from silicon instead of the traditional PDMS. The chip’s unique design includes an asymmetrical microchannel with a silicon nanoblade on one side of the deformation zone. This structure applies contact pressure to CD34 + HSPCs, disrupting their membrane for efficient macro-molecule or plasmid delivery. **d**. An Illustration of the Electroporation Process. This image demonstrates the electro-physical technique of electroporation, a swift and non-viral method used for the delivery of foreign substances into cells. The process involves the disruption of the cell membrane’s phospholipid bilayer through the application of an electric field, resulting in temporary pores that allow the ingress of external molecules. **e**. The Filtroporation Process. This illustration demonstrates the operation of filtroporation devices, a technology that permeabilizes cells by drawing them through the pores of a cell culture insert using a vacuum. The image provides a visual representation of how this technology facilitates the passage of cells through these pores, effectively permeabilizing them. **f**. Cell-Penetrating Peptides (CPPs). This illustration depicts the process by which CPPs, short peptide fragments, transport various molecular cargos across the cellular membrane through endocytosis, serving as a molecular delivery vehicle. (This figure was created by the authors using Adobe Photoshop software)

Achieving efficient delivery of RNPs into target cells is a significant hurdle for their widespread utilization. The intricate composition and charge properties of RNPs present specific challenges when contrasted with the delivery of proteins or nucleic acid systems [47]. Delivery approaches for Cas9 Ribonucleoprotein can be broadly categorized into physical (carrier-independent) and carrier-dependent methods. While physical methods are reliable, they lack specificity and scalability. These methods are more uncomplicated than alternative non-viral approaches for transporting CRISPR/Cas9 cassettes. They employ physical forces to disrupt host cellular and nuclear membranes, facilitating the intracellular delivery of CRISPR/Cas9 components. Widely used for transfecting nucleic acids into challenging-to-transfect cells, these physical delivery approaches, including electroporation, nucleofection, and mechanical transfection, have been applied to introduce CRISPR/Cas9 RNP into HSCs. While these techniques are simple and reproducible, challenges arise in handling bulk cell populations, resulting in heterogeneous responses within the cell population [48]. In contrast, carrier-dependent delivery offers advantages such as scalability, specificity, biodegradability, high packaging capacity, ease of fabrication, and biostability [49] (Tables 1 and 2; Fig. 2).

**Table 1:** CRISPR RNP Delivery systems for HSCs

Delivery method	Condition	Cargo/ target	Intervention /Technique	Clinical trial registry number	Reference
**Elecetroporation**	β-Thalassemia	Cas12a- RNP targeting γ-globin promoter	EDIT-301	NCT05444894	-
	Sickle cell disease	Adenine base editor RNP/ activation of fetal Hb	BEAM-101	NCT05456880	-
	Sickle cell disease	Cas9 RNP + ssODN targeting HBB gene	CRISPR-SCD001	NCT04774536	-
	Sickle cell disease	Cas9 RNP + AAV6 targeting HBB	GPH	NCT048119841	-
	Acute myeloid leukemia	Cas9 RNP targeting CD33	VOR33	NCT 04849910	-
	β-Thalassemia Sickle cell disease	Cas9 RNP targeting BCL11A gene	CASGEVY (CTX-001)	FDA -Approved (NCT03655678)	-
**Microfluidic-Based Methods**	Acute myeloid leukemia	Cas9 RNP targeting CEBPα/CEBPA	Nano-blade chip specifically for HSPC	-	[66]
**Filtroporation**	-	Cas9 RNP targeting CD55 gene	Fluorinated Silane-Modified Filtroporation Devices	-	[68]
	-	Cas9 RNP targeting β2-M and HBG gene	Transmembrane internalization assisted by membrane filtration (TRIAMF),	-	[69]
**Polymer nanoparticles**	-	Cas9 RNP targeting CD33 gene	PBAE-based polymer nanoparticle	-	[88]
	-	Cas9 RNP targeting HBG1/HBG2	PLGA- nanoparticles	-	89
**Cell penetrating peptides**	-	Cas9 and Cas12a-RNP targeting BCL11A	Peptide-Assisted Genome Editing (PAGE) CRISPR–Cas	-	93

**Table 2:** Comparison of methods for delivering RNP to HSCs

Method	Advantages	Disadvantages
**Electroporation** **Nucleofection**	• Efficient delivery of RNPs into HSCs • Simple and widely used • Suitable for ex vivo applications (gene editing in isolated HSCs) • High transfection efficiency • Compatible with primary cells and hard-to-transfect cell lines	• Requires specialized equipment and expertise • May cause cell damage and reduce cell viability • Requires optimization for specific cell types • Potential cytotoxicity and cell stress
**Filter-based methods (Filtroporation )**	• Scalable and amenable to high-throughput applications • Minimal impact on cell viability	• Limited control over delivery kinetics • May not be suitable for all cell types
**Microfluidic-based methods**	• Precise control over fluid flow and mixing • Reduced reagent consumption • Reduced toxicity compared to electroporation/nucleofection	• Complex device fabrication and operation • Limited throughput for large-scale applications
**Nanotechnology-based methods**	• Enhanced cellular uptake and stability of RNPs • Potential for multifunctional nanoparticles (simultaneous drug delivery)	• Challenges in achieving tissue-specific targeting • Safety concerns related to nanoparticle toxicity
**Cell-penetrating peptides**	• Direct cytoplasmic delivery of RNPs • Minimal impact on cell viability	• Variable efficiency depending on peptide sequence and cargo • Challenges in CPP design and optimization • Potential off-target effects due to non-specific uptake

### Electroporation and nucleofection

Electroporation is a rapid and non-viral electrophysical method utilized to deliver exogenous materials into cells and tissues. It involves applying an electric field to disturb the membrane’s phospholipid bilayer, creating temporary pores that enable the introduction of external molecules into cells. While electroporation is considered safer and more cost-effective than viral methods, suboptimal optimization may lead to cell death, particularly in stress-sensitive cells [50]. This technique enables transient and stable transfection of RNPs in various cell types, including human CD34 + hematopoietic stem/progenitor cells (HSPCs) [32, 33, 51–53], human embryonic stem cells (hESCs) [54], human induced pluripotent stem cells (iPSCs) [55], human B cells [56], human CD4 + T cells [57], CAR-T cells [58], and many other cells.

In electroporation, cells suspended in an electroconductive buffer are introduced into a cuvette between two electrodes. Controlled electric pulses with optimized voltages and widths are then applied to create transient pores in the plasma membrane, facilitating the passive influx of impermeable plasmid DNA. This highly efficient delivery approach can enhance exogenous DNA uptake and expression levels up to 1000 times. However, optimal delivery conditions depend on various factors, including electric field characteristics, electrode geometry, and cell and cargo types. Unfortunately, the lack of a one-size-fits-all approach has limited the clinical application of cuvette-based electroporation, and its use is hindered by high costs and the need for expensive reagents and kits [59, 60].

Ongoing clinical studies are exploring the application of electroporation for CRISPR-based gene editing in blood disorders. Notably, the first FDA-approved CRISPR-Cas9 gene therapy, CASGEVY, developed by Vertex Pharmaceuticals and CRISPR Therapeutics for sickle cell disease and β-thalassemia treatment, relies on electroporation for the delivery of RNPs to hematopoietic stem cells (HSCs). CASGEVY (CTX001 Clinical Trial, NCT03655678 and NCT03745287) achieves enhanced expression of fetal hemoglobin (HbF) by disrupting of BCL11A gene in HSCs isolated from the patient’s bone marrow through electroporation of a synthetic guide RNA (gRNA) and *Streptococcus pyogenes* Cas9 protein in the laboratory. Furthermore, a modified version of electroporation known as Nucleofection, designed for the direct delivery of nucleic acids into the nucleus of various cells, has demonstrated efficacy in transfecting human CD34 + cells [61]. While effective, electroporation and Nucleofection may induce cellular toxicity due to transient membrane disruption and potential non-reversible permeabilization. Careful voltage and exposure duration optimization is essential to minimize toxic effects on cells [62].

In one of the recent studies investigating the effect of electroporation on cells, Vavassori et al. examined the impact of electroporation on CD4 + T cells. They studied various groups, including untreated cells, mock-electroporated cells, cells electroporated with RNP without AAV or with an AAV6 donor, and AAV6-transduced cells (without electroporation). Notably, they observed a significant increase in apoptotic/necrotic cell numbers in the mock-electroporated group compared to the untreated group, affecting up to 50% of the cells. Additionally, gene expression analysis revealed the downregulation of genes related to cellular metabolism and the cell cycle in this group, along with the upregulation of genes associated with apoptosis and inflammation. While the presence of the RNP complex may slightly exacerbate these conditions, the toxicity resulting from electroporation is the primary factor contributing to these effects [63].

### Microfluidic-based methods

Microfluidics is a scientific method that controls minute volumes of fluids within channels that are only a few micrometers in size. As cells traverse these microfluidic pathways, they undergo swift mechanical changes [64]. This results in temporary pores in the cell membranes when the combined compressive and shear forces surpass the stress threshold of the phospholipid bilayer. These openings allow biomolecules to diffuse into the cytoplasm passively. The microfluidics approach offers the benefit of high-capacity delivery of nearly all macromolecules into a broad range of cells. This technique has been employed to deliver Cas9 RNP into cells, facilitating genome editing [65].

In 2017, Ma and colleagues pioneered the development of a unique microfluidic chip, the Nano-Blade Chip (NB-Chip), specifically designed for hematopoietic stem and progenitor cells (HSPCs). This innovative chip was constructed using silicon, a departure from the commonly used polydimethylsiloxane (PDMS). The NB-Chip features an asymmetrical microchannel with a silicon nanoblade structure on one side of the deformation zone. This design induces contact pressure on the CD34 + HSPCs, disrupting the membrane and facilitating the efficient delivery of macromolecules or plasmids. Notably, this method proved more effective than electroporation, as it preserved the inherent pluripotency of HSPCs for a longer duration. The researchers successfully delivered the CRISPR in RNP complex format to human HSPCs and disrupted the CCAAT/enhancer-binding protein-α (CEBPα/CEBPA) p42 in vitro. This mutation target is known for inducing acute myeloid leukemia (AML) in which myeloid progenitor proliferation is uncontrollable but the differentiation ability is blocked. The design optimization of the nano-blade structure significantly increased its stiffness and sharpness, further enhancing the delivery efficiency of biomaterials. Notably, the HSPCs treated with this method demonstrated long-term viability and retained their inherent multipotency [66].

### Filtroporation

Over the past decade, a promising biophysical method for intracellular delivery has been developed by Jensen, Langer, and their colleagues, among others. This method involves forcing cells through constrictions that are 30 − 80% of their diameter, which has been found to temporarily permeabilize the cells, making them open to cargo uptake [67]. Filtroporation is a technique that propels cell suspensions through micropores of uniform size in a filter membrane. This action creates mechanical deformation and temporary openings in the membrane, similar to the process used in microfluidics [47].

A recent study by M. Frost and colleagues introduced a technology for intracellular delivery that can be assembled using materials typically found in research labs. This technology broadens the accessibility of intracellular delivery to researchers and clinicians worldwide, particularly in resource-limited areas. The technology, known as filtroporation devices, permeabilizes cells by pulling them through the pores of a cell culture insert using a vacuum found in biosafety cabinets. With less than $10 worth of materials per experiment, the team demonstrated the delivery of fluorescently labeled dextran, expression plasmids, and RNPs for gene knockout to Jurkat cells and human CD34 + hematopoietic stem and progenitor cells. They achieved up to 40% delivery efficiencies for RNP knockout and cell viability of over 80%. The team discovered that delivery efficiency improved when the filter surfaces were functionalized with fluorinated silane moieties. These devices can process between 500,000 and 4 million cells per experiment. When used with a 3D-printed vacuum application chamber, the throughput can be increased 6 − 12-fold in parallel experiments [68].

In another study in 2018, a filtroporation device was described as Transmembrane internalization assisted by membrane filtration (TRIAMF), which is composed of a silicone washer, a stainless-steel mesh, a hydrophilic track-etched polycarbonate filter membrane, and a polytetrafluoroethylene (PTFE) washer. The filter holder is linked to a syringe that acts as a reservoir for RNP and HSC mixture solution. This mixture can be propelled through the filter membrane using nitrogen pressure and then collected into a tissue culture plate. Following the treatment, the expression of β2-microglobulin (β2M) was observed to decrease by 63.1%, with a cell recovery rate of 63.7%. The filtroporation system also resulted in 44% indels on the γ-globin (HBG) gene in HSCs. Furthermore, the filtroporation process did not hinder the multilineage potential and engraftment of HSCs in sub-lethally irradiated non-obese (NOD)/severe combined immunodeficiency (SCID)/Il2rg−/− (NSG) mice [69].

### Nanotechnology-based methods

Therapeutic biomacromolecules, such as DNA and RNA, are prone to degradation in biological fluids. Their hydrophilic nature and negative charge hinder their ability to penetrate the cell membrane [70]. For instance, the sgRNA in an RNP complex carries around 100 negative charges. Despite the Cas9 protein’s 22 positive charges, the RNP complex has a net negative charge, making cellular entry challenging [71]. To address these issues, nanocarriers often deliver CRISPR/Cas9 to target cells. These nano-delivery systems enhance the stability of therapeutic biomacromolecules, shielding them from premature degradation and swift clearance in vivo, thereby facilitating the delivery of their medicinal payload to the target site. Once internalized into target cells via endocytosis and endosomal escape, nanocarriers loaded with Cas9 RNP can reach the nucleus to execute CRISPR/Cas9-mediated genome editing [71].

Lipid-based nanoparticles (LNPs) are carriers containing a uniform lipid core and comprise small molecules, proteins, and DNA. They are increasingly recognized as potential vehicles for delivering a variety of therapeutic agents within the pharmaceutical sector [72]. LNPs offer non-immunogenicity, and biocompatibility, and undergo degradation in the body with minimal to no side effects. They exhibit high hydrophobic and hydrophilic drug encapsulation, enable large-scale production, facilitate controlled and modified release, and enhance drug solubility [73].

In the context of transferring CRISPR-Cas9 system components using LNPs, it should be noted that the Cas9 protein possesses a positive charge (22 net positive charges), preventing it from directly forming a complex with cationic lipids via electrostatic interaction [74]. While some researchers posit that the RNP complex, due to its negative charge, can be delivered using conventional LNPs currently available, the prevailing consensus is that producing novel and optimized LNPs is necessary to achieve the most effective transfer state and maintain high genome editing efficiency [75].

At present, various materials for transfection using LNPs have been commercially produced and are accessible to researchers [76]. Notable among these are Lipofectamine 2000, Lipofectamine 3000, and RNAiMAX, all produced by Thermo Fisher. In response to the need for LNPs optimized for RNP complex delivery, Thermo Fisher introduced the first optimized LNP for RNP complex delivery, Lipofectamin CRISPRMAX Cas9. According to the company, the efficacy of genome editing and target sequence cleavage using this delivery method has been validated in over 20 different cell types, including iPSC, mESC, N2A, CHO, A549, HCT116, HeLa, and HEK293.

Numerous studies have explored the use of lipid nanoparticles to deliver RNP complexes to various cells and tissues, including HEK-293T [77], Hepa 1–6 [77], the human skin epidermal layer [78], induced pluripotent stem cells [79], mouse cortical neurons [80], mouse cornea [81], human skeletal muscles [82], and porcine embryos [83]. However, it seems that the delivery of CRISPR-Cas9 system components as RNP complexes to hematopoietic stem cells using lipid nanoparticles remains unexplored. The FDA has approved the delivery of mRNA via lipid nanoparticles in three therapeutic platforms, namely the COVID-19 vaccines produced by Moderna and BioNTech/Pfizer, and the Patisiran drug, the first siRNA-based treatment to receive FDA approval, for treating polyneuropathy [84]. Consequently, the delivery of the CRISPR-Cas9 system as Cas9 mRNA and sgRNA to hematopoietic stem cells has been the subject of various studies, particularly to apply in vivo gene therapies [85].

In a separate study by Walther et al., the efficiency of delivering CRISPR-Cas9 system components as RNA and as RNP using lipid nanoparticles was examined in HEK293T and Hepa 1–6 cell lines. The study found that Cas9 mRNA incorporates more effectively into the core of the lipid nanoparticle, while the RNP complex tends to associate with the nanoparticle’s outer surfaces. Despite the net negative charge of Cas9-RNP, the negative charges are not uniformly distributed on the complex’s surface, leading to less effective encapsulation in the lipid nanoparticle. [77]

Vavassori et al. compared the efficiency of two delivery methods in genome editing of HSCs, Cas9 mRNA and sgRNA via lipid nanoparticles and RNP via electroporation [63]. Although the genetic editing efficiency, which involved knocking out the B2M gene in this study, is lower in the RNA lipid nanoparticle case than in the high-dose RNP transferred by electroporation, the number of edited cells in both cases is similar. Notably, lipid nanoparticle-based transfer enhances hematopoietic stem cells’ survival and colony-forming ability. However, it is worth noting that since Cas9 mRNA increases the incidence of off-target events compared to RNP, lipid nanoparticles that facilitate the transfer of Cas9-RNP need further refinement.

Polymer nanoparticles (PNPs) are another category of nanoparticles that have been used to deliver RNP complexes. Based on the evidence obtained from conducted studies, using polymeric structures to deliver CRISPR-Cas9 system components has been safe and efficient, and even the speed, timing, and location of delivery can be controlled using this method [86].

So far, a few polymeric systems have been developed for the delivery of genome editing systems, including boronic dendrimer, Cas9 micelles, and polymeric nanocapsules [87]. However, it should be noted that using polymer nanoparticles, like LNPs, to deliver RNP to HSCs has only been investigated in a few limited studies. El-Kharrag et al. conducted a study in 2022 that examined the delivery of RNP using PNPs to HSC. In this study, the RNP complex, which targeted the CD33 gene, was delivered to GCSF- mobilized CD34 + cells using a PBAE-based polymer nanoparticle. After the delivery of the PNP-based RNP complex to CD34 + cells, cell survival was over 86%, and the reduction in CD33 gene expression varied from 13 to 85%. Then they compared the reduction in CD33 expression, cell survival rates, and long-term multilineage engraftment potential of delivering RNP using polymer nanoparticles and electroporation. Cell survival in the electroporated group was 72% and in the nanoparticle group was 85%. Regarding the reduction in CD33 expression, 88% and 76% reductions were seen in the nanoparticle group and electroporation, respectively. Finally, concerning graftability and multilineage differentiation potential, human chimerism in the electroporated group was three to five times less than in the nanoparticle group. [88]

In another study in 2021, a biodegradable polymer nanoparticle with FDA approval named PLGA was used to deliver the RNP complex to HSPC and the HUDEP-2 cell line. Initially, RNP-PLGA-NPs were delivered to the human umbilical cord blood-derived erythroid progenitor-2 (HUDEP-2) cell line, transduced with lentiviruses expressing eGFP. The sgRNAs of this complex targeted the eGFP gene, and 13 days after the delivery of the complex, GFP expression in HUDEP-2 cells decreased by up to 70%. After confirming the efficiency of RNP-PLGA-NPs in the cell line, the researchers transferred this complex to HSCs. At this stage, sgRNAs were used to target the gamma-globin gene promoter (the site of action of gamma gene expression inhibitors). By analyzing the Indels created after editing, about 40% of BFU-E colonies had Indel mutations in the target locus. All analyzed colonies were mosaic for HBG1/HBG2 mutations, indicating that after the initial burst release, the CRISPR components were released slowly and remained functional during the proliferation of methylcellulose colonies [89]. This establishes CRISPR/Cas9-PLGA-NPs as an efficient non-viral delivery system for CRISPR/Cas9 to HSPCs.

### Cell penetrating peptides

Cell-penetrating peptides (CPPs), short peptide fragments, can transfer diverse molecular cargos across the cellular membrane via endocytosis, functioning as a molecular delivery vehicle [90]. The transactivating transcriptional activator (TAT) was the first CPP discovered in the Human Immunodeficiency Virus (HIV-1). TAT possesses a cationic peptide sequence comprising 11 amino acids, which is instrumental in facilitating intracellular delivery [91].

Apart from CPPs that are protein-derived, synthetic peptides that bear chimeric sequences originating from two disparate proteins can be employed to expedite intracellular delivery [92]. It has been documented that CPPs can mediate the delivery of functional molecules, notably the RNP complex, into HSPCs (70).

Zhang and colleagues developed a Peptide-Assisted Genome Editing (PAGE) CRISPR–Cas system that allows for easy, efficient, and non-harmful genome editing of primary cells [93]. The PAGE system comprises a cell-penetrating Cas protein, such as Cas9 or Cas12a, and a cell-penetrating endosomal escape peptide. After a brief incubation period of just 30 min, the PAGE system can perform strong gene editing in both Cas protein and Cas RNP complex formats, while causing minimal cellular toxicity and disruption of gene transcription. The team demonstrated the effectiveness of the CRISPR-PAGE system for highly efficient single and multiplex genome editing in human primary T cells and hematopoietic progenitor cells [93]. After confirming this system’s effectiveness in CAR-T cells, they evaluated its performance in HSCs. For this purpose, they selected the BCL11A gene, the main factor inhibiting the expression of the gamma-globin gene, as the target for destruction by the CRISPR system. They calcified HSCs in two groups: in the first group, HSCs were edited with Cas12a-RNP-PAGE and the second group, was edited with the conventional method, i.e., RNP electroporation. These two groups were compared regarding genome editing efficiency, cell expansion, and HbF activation after differentiation into the erythroid lineage. Based on the TIDE indel analysis, both groups achieved approximately 100% efficiency in editing the target area, i.e., BCL11A + 58 kb enhancer. After six days of expansion, HSPCs edited by PAGE produced approximately three times more erythroid cells than electroporated cells, suggesting that PAGE editing is less harmful to cell viability than electroporation. Flow cytometry further showed that cells edited by opCas12a-RNP-PAGE resulted in increased HbF + cells with therapeutically relevant levels of HbF production. Together, these data support the use of opCas12a-RNP-PAGE as a simple and robust approach for ex vivo hematopoietic progenitor cell gene editing and provide a strong rationale for generalizing the CRISPR-PAGE platform for genome engineering of primary hematopoietic lineage cells [93].

## Conclusion

The successful delivery of CRISPR-Cas RNPs to hematopoietic stem cells is not just a technical challenge, but a pivotal factor that could redefine the boundaries of genetic medicine. The methods discussed in this paper - electroporation, microfluidics, Filtroporation, and nanotechnology-based methods - each represent a unique approach to this challenge, offering diverse strategies to overcome the barriers to effective RNP delivery. Electroporation, which uses an electric field to increase cell membrane permeability, has been a mainstay in molecular biology for decades. However, its application in delivering RNPs to hematopoietic stem cells requires careful optimization to prevent cellular damage and maintain cell viability. Microfluidic systems, on the other hand, offer a high degree of control over the delivery process, potentially improving the precision and efficiency of RNP delivery. However, the technical complexity of these systems may pose challenges for their widespread adoption. Filtroporation, a newer technique, shows promise for enhancing delivery efficiency by using a filter to create temporary pores in the cell membrane. While promising, more research is needed to fully elucidate this method’s potential and limitations. Nanotechnology-based methods represent an exciting frontier in RNP delivery. By packaging RNPs into nanoparticles, these methods offer the potential for targeted delivery and reduced off-target effects. However, the development of these techniques is still in its early stages, and much work remains to be done to realize their full potential.

This review has explored several methods of RNP delivery, each with its unique advantages and challenges. However, it is essential to note that some methods, such as extracellular vesicles, exosomes, microinjection, and induced transduction by osmocytosis and propanebetaine (iTOP), have not been extensively explored in the context of delivering RNPs to hematopoietic stem cells. While promising in other applications, these methods may present unique challenges or opportunities in the context of hematopoietic stem cells that warrant further investigation.

As the field of gene editing continues to advance, it is crucial to continue refining these delivery methods and developing new ones. The ultimate goal is to achieve safe, efficient, and targeted delivery of CRISPR-Cas RNPs to hematopoietic stem cells, thereby unlocking the full potential of this powerful gene-editing tool for therapeutic applications.

## Data Availability

Not applicable.

## Abbreviations

HSC: Hematopoietic Stem Cells
CRISPR: Clustered Regularly Interspaced Short Palindromic Repeats
RNP: Ribonucleoprotein
ZFN: Zinc Finger Nucleases
TALEN: Transcription Activator-Like Effector Nucleases
DSBs: Double-Strand Breaks
crRNA: Crispr Rna
tracrRNA: Transactivating RNA
sgRNA: Single Guide RNA
NHEJ: Nonhomologous End-Joining
HDR: Homology-Directed Repair
HSCT: Hematopoietic Stem Cell Transplantation
SCD: Sickle Cell Disease
HLA: Human Leukocyte Antigen
GVHD: Graft Versus Host Disease
NB-Chip: Nano-Blade Chip
PDMS: Polydimethylsiloxane
CEBPα: CCAAT/Enhancer-Binding Protein-Α
AML: Acute Myeloid Leukemia
TRIAMF: Transmembrane Internalization Assisted By Membrane Filtration
PTFE: Polytetrafluoroethylene
β2M: Β2-Microglobulin
LNPs: Lipid-Based Nanoparticles
PNPs: Polymer Nanoparticles
CPPs: Cell-Penetrating Peptides
TAT: Transactivating Transcriptional Activator
PAGE: Peptide-Assisted Genome Editing
